# A 6-Month mHealth Low-Carbohydrate Dietary Intervention Ameliorates Glycaemic and Cardiometabolic Risk Profile in People with Type 2 Diabetes

**DOI:** 10.3390/nu17060937

**Published:** 2025-03-07

**Authors:** Despina Kolivas, Liz Fraser, Ronald Schweitzer, Peter Brukner, George Moschonis

**Affiliations:** 1 School of Allied Health, Human Services & Sport, La Trobe University, Bundoora 3086, Australia; 2 Watson General Practice, 34 Windeyer Street, Watson 2602, Australia; 3 East Bentleigh Medical Group, 873 Centre Road, Bentleigh East 3165, Australia; 4 Department of General Practice, School of Public Health and Preventive Medicine, Monash University, Level 5, 553 St Kilda Rd, Melbourne 3004, Australia; 5 La Trobe Institute for Sustainable Agriculture & Food (LISAF), La Trobe University, Bundoora 3086, Australia

**Keywords:** type 2 diabetes mellitus, low carbohydrate, nutrition, diabetes management, self-management, mHealth, online

## Abstract

**Aim**: Mobile health (mHealth) applications have been reported to be effective in improving glycaemic control and cardiometabolic health, but mainly as part of shorter-term intervention studies. The aim of this study is to examine the effect of the ongoing Defeat Diabetes mHealth low-carbohydrate diet (LCD) intervention on clinical markers and cardiometabolic risk after 6 months of intervention. **Methods**: Data were collected via primary care physicians as part of routine T2D monitoring. These included HbA1c (primary outcome), blood pressure, blood lipids, and markers of kidney and liver function. Anthropometrics, as well as changes in the prescription of diabetes, hypertension, and dyslipidaemia medication, were also recorded. Calculated variables, total cholesterol to HDL-c, triglyceride to HDL-c, and waist to height ratios, were analysed to examine changes in cardiometabolic risk profile. Three-day food records were used to assess dietary intake and intervention adherence. Univariate regression models examined changes from baseline to 6 months. **Results**: Ninety-four participants remained in the study out of the ninety-nine at baseline (mean age 59 ± 11 years, 55 females). After 6 months of intervention, there were significant reductions in HbA1c by −1.0% (95% CI: −1.3 to −0.6), as well as in the liver enzymes ALT (−9.3 U/L 95% CI −16.3 to −2.4) and GGT (−18.8 U/L 95% CI: −31.4 to −6.3) across the cohort. In addition, there was a significant reduction in cardiometabolic risk, as measured by the calculated variables and a decrease in waist circumference (−4.6 cm 95% CI: −8.9 to −0.2). **Conclusions**: People with T2D receiving LCD education and resources through the Defeat Diabetes mHealth app (version 3.3.8) improved their glycaemic control after 6 months of intervention. Cardiometabolic risk profile and liver function also showed significant improvement. These findings indicate that the use of an LCD digital app is a valuable adjunct in the management of T2D.

## 1. Introduction

Prior findings from an mHealth T2D LCD delivered via the Defeat Diabetes application (app) within the Australian primary care context have shown significant improvements in glycaemic control, systolic blood pressure, and weight status after 3 months of intervention [1]. An LCD is generally defined as one in which less than 26% of energy is derived from carbohydrates (or less than 130 g per day), while a very low-carbohydrate ketogenic diet (VLCKD) is a diet with less than 10% of energy derived from carbohydrates (usually between 20 and 50 g of carbohydrate per day) [2]. The study intervention involved the integration of an LCD digital app that provides education and support, as an adjunct treatment modality in primary care management of T2D. The Royal Australian College of General Practitioners (RACGP) recommends that people with T2D be monitored on a 3-monthly basis, as part of the annual diabetes cycle of care and sets individual goals for T2D management, including glycaemic control (HbA1c ≤ 7%) and lipid management, as well as treatment targets for hypertension and monitoring of kidney function [3,4].

In people with T2D, cardiovascular disease (CVD) is the leading cause of death. As such, risk assessment and prevention strategies, including lifestyle modifications and prescription of medications, are recommended as part of routine monitoring and management [3,5]. T2D, body mass index (BMI), systolic blood pressure, non-high-density lipoprotein cholesterol, and current smoking status are five modifiable risk factors for the development of CVD. Analysis by the Global Cardiovascular Risk Consortium has shown that out of these five modifiable risk factors, T2D has the greatest hazard ratio of incident CVD and death from any cause [6]. In addition, chronic kidney disease (CKD) in people with T2D is the single leading cause of kidney failure [3]. The clinical diagnosis of diabetes-related CKD is characterised by a decrease in kidney function over time, as measured by the estimated glomerular filtration rate (eGFR) in the presence of longstanding T2D [7]. Elevated systolic blood pressure is associated with CKD in people with T2D and as such is a target for risk reduction [3]. In addition, people with T2D have a higher incidence of liver abnormalities, presenting as chronic mild elevations of transaminases, and it is well established that there is an increased risk of incident T2D in people diagnosed with metabolic-associated steatotic liver disease (MASLD), previously referred to as non-alcoholic fatty liver disease (NAFLD) [8,9,10]. Increases in alanine transaminase (ALT) and gamma-glutamyl transferase (GGT) are associated with insulin resistance, metabolic syndrome, and T2D [9,10,11,12].

Prior research on the use of LCDs in T2D, has found significant improvement in glycaemic control, reductions in body weight and medication use, and increased likelihood of diabetes remission, which is defined as maintaining “HbA1c < 6.5% measured at least 3 months after cessation of glucose-lowering pharmacotherapy” [13,14,15,16,17,18,19]. In addition, improvement is seen in several key clinical parameters that underlie cardiometabolic risk in people with T2D using an LCD, both in clinical practice and in academic research [13,14,18,20,21,22,23,24,25,26]. LCDs have also shown promise in improving kidney function, as measured by eGFR, as well as liver function, as measured by ALT and GGT [27,28,29,30,31]. Despite this, the use of an LCD in the management of T2D remains controversial, in part due to the longstanding unfounded belief that dietary fat contributes to obesity and thereby increases cardiometabolic risk [32]. Proxy ratios, including the total cholesterol to HDL ratio and triglyceride to HDL ratio, as well as the waist to height ratio, which are used to determine underlying cardiometabolic risk, do not support this assertion [33,34,35,36,37,38,39,40,41]. The calculation of these proxy ratios can be useful in assessing the trend in cardiometabolic risk over time, and the input parameters are readily available from outcomes measured in routine T2D management at the primary care level.

There are significant clinical implications from the integration of digital apps for use in a primary care setting. Digital tools used for the management of chronic health conditions, such as T2D, provide additional education and support to patients when compared to standard care alone and can lead to better health outcomes [42]. This experimental study design, which incorporates the education of health care providers on the LCD management of T2D, while utilising the existing health care framework for monitoring and data collection, in combination with the use of an LCD app for education and peer support of patients, has not previously been investigated. A longer-term evaluation of the intervention to assess the effects on dietary adherence and sustainability, glycaemic control and changes in cardiometabolic risk parameters, will provide evidence as to the applicability of the intervention in the context of the Australian primary health care setting.

The aim of this study is to examine the ongoing effect of an LCD via the Defeat Diabetes mHealth app on the clinical markers routinely measured and monitored as part of T2D management. We seek to understand whether there is a sustained improvement in glycaemic profile, blood pressure, and anthropometric markers following 3 months of intervention and to assess changes in lipid profile, renal function, and liver function after 6 months of intervention.

## 2. Materials and Methods

### 2.1. Study Design and Participants

A comprehensive study design protocol previously detailed the methodology [43]. This single-arm pre–post study design follows participants over a 12-month period. The results presented in the current paper represent data collected after 6 months of follow-up. The results presented after 3 months of follow-up are published elsewhere [1]. Participant data were collected over the period from October 2022 to July 2024.

As medical monitoring of participants is required for the duration of the intervention, only participants referred via registered supporting GPs were eligible to participate. The study was advertised via GP networks, and GPs who were interested in providing support registered with the research team, who provided detailed study information and patient handouts to assist with recruitment. The primary inclusion criteria were HbA1c ≥ 6.5%, access to a smartphone or PC, and ability to use digital apps. Major inclusion/exclusion criteria are outlined elsewhere [43].

All people deemed eligible to participate provided their informed consent for inclusion before participation in the study. Data collection was facilitated by online research electronic data capture (REDCap) case report forms (CRFs) sent via email to GPs and participants [44,45].

Approval to conduct the study was granted by the La Trobe University Human Research Ethics Committee (HREC) (approval no. 22117, 11 July 2022). The trial was registered with the Australian New Zealand Clinical Trials Registry (ANZCTR) on 17 May 2022, with the ACTRN: 12622000710729.

### 2.2. Study Intervention

After informed consent and baseline data were obtained, the participants were granted access to the Defeat Diabetes mHealth app and asked to follow the program’s instructions over the course of the next 12 months.

The Defeat Diabetes mHealth app is a subscription-based commercial app for download on a smartphone (Android and Apple iOS) or use in a web browser and provides a guided educational program on carbohydrate reduction and lifestyle interventions to manage T2D https://www.defeatdiabetes.com.au/ (accessed on 3 February 2025) [46].

On registration confirmation, the participants were sent a series of emails from Defeat Diabetes explaining how to use the app. They were instructed to follow the video lessons in a sequence. The Defeat Diabetes mHealth app provides low-carbohydrate recipes and cooking demonstration videos, meal planning, shopping lists, and exercise plans, as well as a comprehensive recommended food list with a rating system to guide food choices. The Defeat Diabetes mHealth app also encourages users to participate in a moderate amount of physical activity to assist with more effective glycaemic control.

Additional support is provided to users with the option to join a private Defeat Diabetes Community Facebook group. App news and events such as the live and recorded webinars are disseminated via a weekly email newsletter.

## 3. Outcomes

GP monitoring of patients with T2D is subsidised as part of the Australian Government’s universal health insurance scheme, Medicare [47]. The outcomes reported are based on the medical examinations covered under this scheme, and where they are not, they are optional. All blood samples are collected and analysed at a clinical pathology laboratory (as ordered by each GP), and other measurements are taken on site at each GP practice.

### 3.1. Primary Outcome

The primary outcome in the present study was the change in HbA1c from the data provided by the participants’ GPs at baseline and after 3 and 6 months of follow-up.

### 3.2. Secondary Outcomes

The participants’ GPs also provided data related to secondary outcomes, including fasting plasma glucose (FPG), systolic and diastolic blood pressure, lipid biomarker total cholesterol (TC), low-density lipoprotein cholesterol (LDL-c), high-density lipoprotein cholesterol (HDL-c), serum triglycerides (TRIG), and renal function (calculated eGFR). There was the optional provision for the analysis of specific blood biomarkers, including liver enzymes, alanine transaminase (ALT), and gamma-glutamyl transferase (GGT), as well as inflammatory markers (hs-CRP, CRP). The GPs also provided anthropometric markers—body weight, body mass index (BMI), and waist circumference (WC). Calculated variables, such as total cholesterol to HDL-c, triglyceride to HDL-c, and waist to height ratios, were also analysed [35,37,41,48,49,50]. Prescription medication use for diabetes, hypertension, and dyslipidaemia was recorded at baseline, at 3 months, and at 6 months. Adverse events reported by supporting GPs or participants during the follow-up period were reported to the principal investigator and reviewed by the La Trobe University HREC.

### 3.3. Participant Baseline Characteristics

Baseline demographic characteristics included age, sex, country of birth, educational history, and coexisting medical conditions, specifically those that may be related to any of the outcome measures. In addition, the time of T2D diagnosis was recorded, as the recency of diagnosis may be associated with increased likelihood of T2D remission [19,30]. The number of other people living in the participant’s household was also recorded, as this may provide some background into potential barriers or enablers in the success of the intervention and may reflect the level of support participants receive from family members.

### 3.4. Dietary Intake and Adherence to the Intervention

Three-day food records were completed by the participants and submitted via the online REDCap data collection submission forms, via email or via text message to the research team. Data from the food records were entered into FoodWorks Professional 10, Brisbane, Queensland, Australia (Version: 10.0.4266) (2020), and a dietary analysis was obtained at baseline and after 3 months [51]. When a participant was unable or unwilling to complete a 3-day food record, the research team provided the option of a 24 h dietary recall over the phone, following a standardised process by a trained nutritionist. The dietary data served to elucidate the changes in carbohydrate intake and adherence to the intervention.

### 3.5. Impact of Physical Activity

Physical activity levels were monitored using the short version of the International Physical Activity Questionnaire (IPAQ) [52], which records physical activity levels during the previous 7 days. The IPAQ was completed by participants in a self-administered format. The classifications for physical activity as reported by the IPAQ include high, moderate, and low. A high level of physical activity equates to more than one hour of moderate intensity physical activity per day. A moderate level of physical activity equates to some activity (about half an hour) of at least moderate intensity physical activity on most days. A low level of physical activity implies that the above criteria are not met.

### 3.6. Statistical Analyses

Continuous variables were examined for the normality of their distribution using the Kolmogorov–Smirnov test. Univariate linear regression models were used to assess within-group changes in all continuous study outcomes from baseline to the 3-month follow-up. These regression models were adjusted for appropriate covariates, which included sex and age. Changes in the categorical variables from baseline to the 6-month follow-up were tested using the chi-square test. The statistical analyses were conducted for the total sample and, after stratification, by gender.

All statistical analyses were performed using SPSS statistical software for Windows (Version 28.0, Armonk, NY, USA). All reported *p* values were two-tailed, and the level of statistical significance was *p* < 0.05.

## 4. Results

After 6 months, a total of 94 participants remained active in the study as per the protocol.

**Figure 1:**
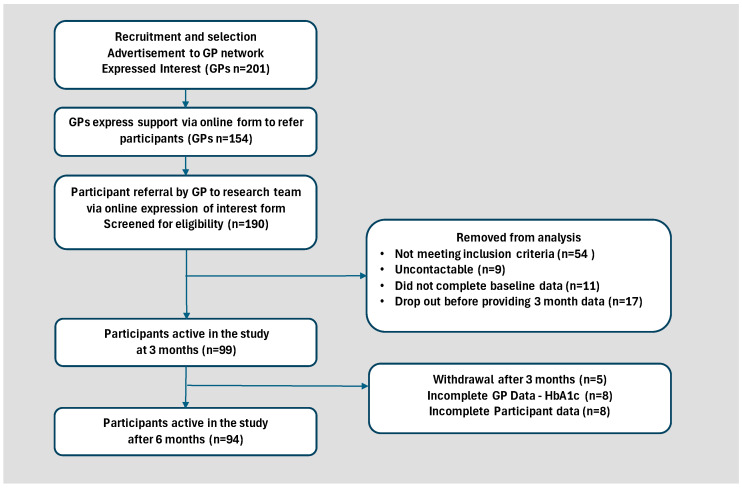
Study process and recruitment.

Figure 1 depicts the study flow from recruitment to the 6-month follow-up.

### 4.1. Participant Baseline Characteristics

Table 1 describes the socio-demographic characteristics of the study participants in the total sample (*n* = 99). The median time since diabetes diagnosis was 2.9 years, with approximately 30% of participants diagnosed within the previous year and others having diabetes for more than 20 years. Seventy-eight percent of the study participants reported having one or more co-existing medical conditions. These included cardiac issues (15%), gastrointestinal disorders (5%), hyperthyroidism or metabolic bone disease (1%), osteoporosis (5%), rheumatoid arthritis (4%), psychological disorders (9%), hypertension (57%), high blood cholesterol (42%), prior gastric bypass surgery (2%), significant kidney or liver disease (4%), and immunodeficiency (2%). According to the classifications specified by the International Physical Activity Questionnaire (IPAQ), approximately 41% of the participants were moderately active, while approximately 26% and 32% were classified as having low or high levels of physical activity, respectively [52].

### 4.2. Changes in Dietary Intake

The changes observed in dietary energy and macronutrient intake from baseline to follow-up, are summarised in Table 2. After 6 months of intervention, a significant decrease in average total energy intake (−1301 kJ/day, 95% CI: −1917 to −685) was observed in both males (−1249 kJ/day, 95% CI: −2201 to −297) and females (−1243 kJ/day, 95% CI: −1981 to −504).

The participants’ macronutrient intake, expressed as proportion of energy intake, changed significantly from baseline to the 6-month follow-up, with a significant reduction in dietary carbohydrate intake (−14% kJ/day, 95% CI: −17 to −11) and significant increases in protein (6% kJ/day, 95% CI: 4 to 8) and total fat (9% kJ/day, 95% CI: 6 to 11) intake. As can be seen in Table 2, saturated fat as a proportion of overall energy significantly increased across the whole cohort (4% kJ/day, 95% CI: 3 to 5). There was a significant decrease in polyunsaturated fat as a proportion of fat across the whole cohort (−3% fat/day, 95% CI: −4 to −1). There was no significant change in monounsaturated fat as a proportion of dietary fat after 6 months from baseline. Dietary fibre significantly decreased (−3 g/day 95% CI: −5 to 0) overall in the total sample over the 6-month period. Eight participants did not provide food record data.

### 4.3. Changes in Physical Activity Levels

After 6 months of intervention, there were no significant differences in the percentage of participants allocated to the low, medium, and high physical activity level categories in the total sample and when stratified by gender (data not presented in tables).

### 4.4. Changes in Clinical Outcomes

Table 3 summarises the changes observed in the examined clinical outcomes from baseline to follow-up. These changes are presented for the total sample and are stratified by gender, from baseline to the 6-month follow-up in the examined clinical outcomes. The analyses performed in the total sample showed a significant reduction in HbA1c (−1.0%, 95% CI: −1.3 to −0.6) and FPG (−1.3 mmol/L, 95% CI: −2.0 to −0.6) over 6 months. The changes in HbA1c were more pronounced in males (−1.2%, 95% CI: −1.7 to −0.7) compared to females (−0.8 mmol/L, 95% CI: −1.2 to −0.4). Serum triglycerides showed a significant reduction in the total sample (−0.4 mmol/L 95% CI: −0.8 to −0.1); see Supplementary Table S1. The liver enzymes ALT (−9.3 U/L 95% CI: −16.3 to −2.4) and GGT (−18.8 U/L 95% CI: −31.4 to −6.3) also showed significant reductions, with more pronounced changes in ALT (−10.2 U/L 95% CI: −19.4 to −1.0) and GGT (−17.4 U/L 95% CI: −32.9 to −1.9) in the female participants. Waist circumference showed a significant reduction across the cohort (−4.6 cm 95% CI: −8.9 to−0.2) and specifically in the female participants (−6.3.cm 95% CI: −11.7 to −0.9).

It should also be noted that the ratio of total cholesterol to HDL-c (−0.38 CI: −0.70 to −0.05) was significantly reduced from baseline over the 6-month period. In addition, there were also significant reductions in proxy markers for insulin resistance, as recorded in the triglyceride to HDL-c ratio (−0.50 95% CI: −0.92 to −0.08) in the total sample and the waist to height ratio in the female participants (−0.04 95% CI: −0.07 to −0.01) after 6 months of intervention.

No significant changes were found in total cholesterol, LDL-c, HDL-c, kidney function marker eGFR, systolic and diastolic blood pressure, body weight, or BMI in the total sample and by gender (further details can be found in Supplementary Table S1).

CRP and hs-CRP were requested as optional clinical markers; however, there were insufficient results reported; thus, they were omitted from the analysis.

Eight participants were missing routine bloodwork at the 6-month follow-up for the primary outcome HbA1c.

**Figure 2:**
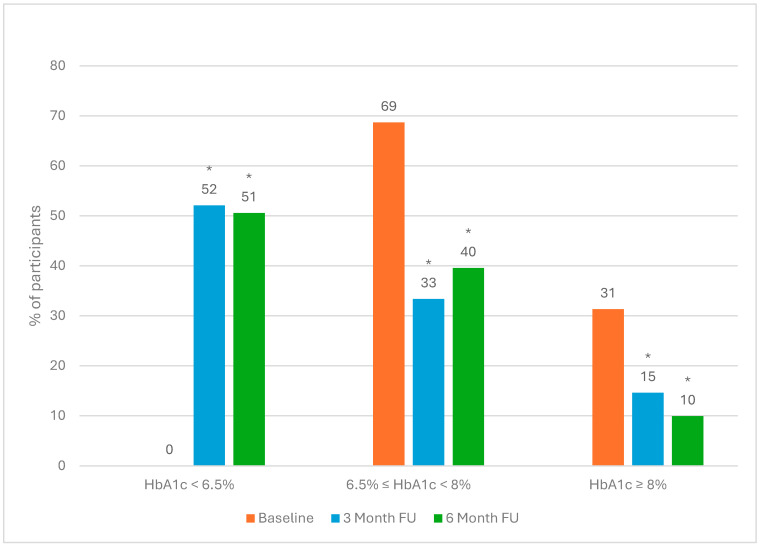
Participants as grouped by HbA1c, less than 6.5%, greater than or equal to 6.5% and less than 8%, and greater than or equal to 8%, at baseline and after 3 and 6 months of intervention. * indicates the statistically significant difference between baseline and follow-up period (*p* < 0.05). Note: All participants at baseline recorded an HbA1c of 6.5% or greater as part of the eligibility criteria.

In addition, Figure 2 shows that 51% of the participants were able to reduce their HbA1c to below the diabetic threshold, defined as achieving an HbA1c of less than 6.5% after 6 months.

### 4.5. HbA1c Reduction Grouped by Weight Loss Category After 6 Months of Intervention

Table 4 shows that the participants in the total sample who lost more than 5% of their initial body weight over the 6 months had a greater reduction in HbA1c compared to those who did not. There was a statistically significant association with reduction in bodyweight and HbA1c in the total cohort (−1.2% ± 1.1% *p* = 0.047) and in the female participants who lost more than 5% of their body weight (−1.2% ± 1.2% *p* < 0.001).

Overall, the majority of the participants (86%) lost weight, with nearly 85% recording a decrease in HbA1c after 3 months of intervention, as shown in Supplementary Table S2.

### 4.6. Adherence to the Defeat Diabetes mHealth App and Its Impact on Glycaemic Control and Weight Loss

Across the cohort, when comparing those whose average carbohydrate intake was 50 g or less with those with an average carbohydrate intake of greater than 50 g, there was a non-significant decrease in HbA1c (−0.7% 95% CI: −1.2 to −0.1), together with a significant reduction in body weight (−4.2 kg, 95% CI: −6.2 to −2.2), directly driven by the female participants, as shown in Table 5. In particular, the participants who reduced their HbA1c were shown to have also significantly reduced their dietary carbohydrates when grouped by intake of 50 g a day or less, compared to those with a higher dietary intake of carbohydrates (*p* = 0.017).

### 4.7. Medication Use Following the Intervention

At baseline, 28 participants were not prescribed any diabetes medication, while 71 participants were prescribed one or more diabetes medications. After 6 months, 19 participants had reduced their diabetes medication dose, with two participants discontinuing all diabetes medication. Ten participants had medication added to their management plan, and three participants had their diabetes medication increased. No change in diabetes medications were recorded after 6 months of follow-up for 44 participants who were prescribed diabetes medications at baseline (and for whom we have data).

At baseline, 53 people were prescribed medication for hypertension, with 46 participants requiring no medication. After 6 months of the intervention, three participants were able to reduce their medication dose, and four people discontinued all hypertension medications. However, two participants had their dose increased, and one participant who was not previously on any hypertension medication had medication added.

At baseline, 55 people were prescribed lipid-lowering medication, with 45 participants requiring no medication. After 6 months of the intervention, three participants were able to reduce their medication dose, and four people discontinued all lipid-lowering medications. However, three participants had their dose increased, and three participants who were not previously on any lipid-lowering medication had medication added.

### 4.8. Adverse Events

Adverse events reported from baseline to the 3-month follow-up period are reported elsewhere [1]. Between the 3- and the 6-month follow-up periods, a total of 10 participants sought medical attention, including 6 who were admitted to hospital. The reasons for seeking medical attention, as stated by the participants, included work-related stress, syncope, emergency eye surgery, COVID-19, cystoscopy, musculoskeletal pain management, impacted bowel, urinary tract infection, and eczema.

### 4.9. Participant Withdrawals Between 3- and 6-Month Follow-Up Periods

Five participants withdrew from the study between the 3- and 6-month follow-up periods. For three of these participants, clinical data were retrospectively collected from their GPs.

## 5. Discussion

We conducted a single-arm pre–post study that aimed to examine the effect of an mHealth low-carbohydrate dietary intervention with the primary outcome being the impact on glycaemic control, as measured by HBA1c, in people with T2D. Glycaemic control was improved and maintained over the 6-month period, with a significant reduction in HbA1c across the whole study cohort from baseline; this was comparable to the reduction in HbA1c reported after 3 months of intervention, thus confirming the sustainability of the benefits of this approach for the glycaemic control of people with T2D for longer than 3 months [1]. Furthermore, it should also be noted that there was no significant change in physical activity levels, as determined by the IPAQ questionnaire. This highlights that the changes observed in HbA1c were more likely due to the change in dietary intake.

Our results are also consistent with the findings of a recent Australian randomised controlled trial (RCT) using a web-based LCD for T2D (the T2Diet) in comparison with standard care alone over a 4-month period [53]. The T2Diet recommends consumption of between 50 and 100 g of carbohydrate a day, with the focus being on non-starchy vegetables and unprocessed nutrient-dense sources of carbohydrate. The T2Diet also prioritises the consumption of mono- and polyunsaturated sources of dietary fat and low-fat dairy products, which is in contrast with the Defeat Diabetes app which recommends the consumption of unprocessed sources of fat, primarily saturated or monounsaturated, and full-fat dairy products only. Despite these differences, the T2Diet intervention group reduced HbA1c by 0.6%, body weight by 3.3 kg, and BMI by 1.1 kg/m^2^ and reduced the use of diabetes medications [53].

As per our previous findings reporting the effect of the intervention at 3 months, a reduction in dietary carbohydrate and overall energy intake by the study participants was maintained at 6 months [1]. It is worth noting that dietary fat is not a primary determinant of body fat, while higher protein diets have been associated with lower glycaemic levels in people with T2D [32,54]. It is likely that increasing levels of protein and fat while lowering the proportion of carbohydrate consumed results in higher levels of satiety and consequently a reduction in energy intake [55,56]. After 6 months, there was a significant decrease in dietary fibre consumed by participants; this is consistent with prior research utilising a self-determined low-carbohydrate dietary approach, which has also shown reduction in dietary fibre over time [57]. However, while this reduction in mean fibre intake per day is statistically significant, it is unlikely to have any clinical implications as the average intake per day decreased from approximately 21 g to 19 g over the 6-month time period.

Furthermore the study showed non-significant reductions in total cholesterol and LDL-c, an increase in HDL-c, and a significant decrease in triglycerides (−0.4 mmol/L) across the cohort, indicating a trend towards a more favourable lipid profile for CVD risk [58,59,60]. These improvements are consistent with those of an RCT comparing the impact of a high-carbohydrate diet versus a low-carbohydrate diet for management of T2D in an Australian setting. This study also found non-significant reductions in total cholesterol and LDL-c, but a significant increase in HDL-c of 0.1 mmol/L and a significant reduction in triglycerides of −0.4 mmol/L over the course of a 12-month intervention period in the low-carbohydrate group [14].

Cardiometabolic risk was also assessed using the total cholesterol to HDL-c ratio. Higher ratios are associated with increased risk of all-cause mortality, particularly the CVD risk [35]. Our results show a significant mean reduction in the total cholesterol to HDL-c ratio (−0.38) after 6 months of intervention. Insulin resistance as measured by the triglyceride to HDL-c ratio significantly decreased (−0.50) across the sample after 6 months of follow-up. This reduction in the triglyceride to HDL-c ratio is consistent with prior outcomes reported on CVD risk factors while following a low-carbohydrate eating approach [61]. In addition, the waist to height ratio is a simple and predictive indicator of health risks associated with obesity and insulin resistance [33,49]. In people with T2D, the waist to height ratio has a stronger association with CVD risk compared to other anthropometric markers [34]. Our results demonstrate a reduction in waist to height ratio across the cohort and a significant reduction for female participants (−0.04) after 6 months, thus also indicating a reduction in CVD risk.

The current study also reported significant reductions in the liver enzymes ALT and GGT, indicating an improvement in liver function. Significant reductions in mean ALT were seen in the overall sample (−9.3 U/L), largely driven by the mean reductions reported for the female participants (−10.2 U/L), while significant reductions in mean GGT were seen in both the males (−21.4 U/L) and females (−17.4 U/L). T2D is associated with disturbed liver function and elevations in liver enzymes [12]. Given that MASLD is strongly associated with insulin resistance, improvement in glycaemic control is the likely mechanism for the improvement in ALT and GGT [10]. Our findings are consistent with prior research on the use of a low-carbohydrate eating approach for management of T2D, showing that reductions in ALT and GGT are indicative of improvements in liver function [62,63].

There was a non-significant trend towards improvement in systolic and diastolic blood pressure after 6 months of intervention. In the 3-month follow-up, we reported a significant improvement in systolic blood pressure across the whole cohort; however, this was not maintained. This is inconsistent with other research that has shown a sustained and significant reduction in blood pressure in people with T2D who follow a low-carbohydrate diet, independent of follow-up duration [64]. There was also no significant change in kidney function, as measured by eGFR after 6 months of intervention. However, we note that the participants increased their dietary protein with no detrimental effect on kidney function. This is consistent with prior research on the impact of protein consumption and kidney function [65]. While many believe that higher protein intakes are harmful to kidney function, emerging research has found that higher protein intakes have no adverse effects on kidney function and can be beneficial in healthy adults and people with T2D who do not have advanced kidney disease [66,67,68,69]. In addition, epidemiological research has found that for elderly people with CKD, higher protein intake is associated with lower all-cause mortality [70]. It has also recently been hypothesised that excessive refined carbohydrate intake increases the risk of developing chronic kidney disease in healthy adults and that a VLCKD may be a potential management strategy [71,72].

Our prior reported analysis after 3 months of intervention showed there were non-statistically significant reductions in body weight recorded across the cohort, also found in the current study despite the fact that around 45% of the participants achieved a weight loss of at least 5% [1]. The Royal Australian College of General Practitioners (RACGP) recommends that for optimum management of T2D people should reduce body weight by 5 to 10% [3]. This advice is consistent with the results of the current study over this longer time period, with the participants who achieved a 5% or more body weight loss having a significantly greater reduction in HbA1c (mean difference −0.7%) compared to those who did not. In terms of adherence, the participants who consumed 50 g of carbohydrate a day or less, also had a significantly greater reduction in HbA1c (−0.7%) and weight loss (−4.2 kg) compared to those who consumed more than 50 g of carbohydrate a day. This shows that adherence to a lower level of carbohydrate consumption both contributes to the reduction in HbA1c and assists with weight loss. The significant reduction in waist circumference (−4.6 cm) across the cohort is a surrogate marker of reduction in visceral adiposity and liver fat deposition [73,74,75]. In contrast, a waist circumference of greater than 90 cm, together with hypertriglyceridemia as well as MASLD, is known to be associated with a greater degree of visceral adiposity [76,77,78].

A key strength of the study is the use of data to measure specific predictive ratios that can act as proxies for insulin resistance and CVD risk. Calculation of the total cholesterol to HDL-c, triglyceride to HDL-c, and waist to height ratio allows us to confirm that the improvements observed in glycaemic control from directly measuring HbA1c, translate into improved insulin sensitivity and reduction in CVD disease risk. Additional study strengths have previously been outlined, including online coordination of the study protocol allowing a wider reach and facilitation of the data collection process via online REDCap CRFs for GPs and study participants [1]. Costs to administer the study were minimal as the clinical results collected fell within the diabetes annual cycle of care recommendations [3,4]. The intervention also has a relatively low cost in terms of administration and convenient accessibility, compared to the engagement of allied health services. The inclusion of 3-day food records (and, as an option, 24 h dietary recalls) provided an assessment of dietary intake in a standardised manner. Hence, the results and applicability of the findings have high external validity in the Australian context.

As mentioned previously, the study limitations reflected real-world heterogeneity and challenges [1]. Food record data have limitations, with some records collected at baseline showing that a small group of participants had decreased their carbohydrate intake after referral but before providing informed consent to participate, due to their eagerness to commence the LCD approach. It is well understood that food record data are also subject to recall bias and subjectivity. There is also the issue of digital competence and the extent to which people were able to understand and utilise the Defeat Diabetes mHealth app features. In addition, not all participants were Facebook users and thus would not have had access to extensive peer support. In addition to these limitations, it is important to highlight that Australia is a multicultural community and, as such, persons from culturally and linguistically diverse backgrounds with T2D who are not fluent in English, may not have been afforded the opportunity to participate in the study. The Defeat Diabetes mHealth app was developed to help people with T2D in an Australian context and, as such, may require adaptations in order to be applied to other cultures or in other countries. With respect to data collection, some participants had difficulty with scheduling appointments due to travel, work, or the unavailability of their GP. The timing discrepancy reflects the real-world practice of medicine. Some participants did not complete fasting blood tests, and in some cases, there was a significant amount of clinical data missing, such as blood lipids, liver enzymes, kidney function, inflammatory biomarkers, blood pressure, and weight and waist circumference. There are also limitations in our protocol as we did not provide standardised procedures for the recording of anthropometry, weight data, and blood pressure, where results can vary with time of day or technique. In terms of data analysis, it may have been beneficial to include other blood biomarkers of insulin resistance, such as fasting insulin, C-peptide, and fructosamine, to further assess and confirm the impact of the intervention [79,80]. However, these tests are not recommended by the RACGP and, as such, would not be funded by Medicare or normally be requested by GPs in the routine management of T2D. We acknowledge these limitations and that compromises were required to integrate the protocol requirements within the context of the health care system. These compromises allowed greater participation of people with T2D and their GPs in our research working within the scope of usual T2D monitoring. Lastly as previously reported, the limitation in the lack of a control group in our study is ameliorated by subgroup analysis showing that the participants who had greater adherence to the intervention, including those who had the greatest weight loss, had better overall outcomes and reductions in HbA1c, which was maintained over the 6-month follow-up period [1].

Lifestyle interventions depend on many factors and adherence may be impacted by indeterminate external factors. In particular, the application of the intervention may be affected by the underlying motivation and readiness for undertaking lifestyle changes. Understanding motivation and readiness for change may provide us with information as to which participants are more likely to benefit from the intervention [81]. To address these factors, new features that incorporate psychological support for lifestyle change could be added to the app; these may provide additional benefit to users in terms of engagement and sustainability. Future research could include a control group undertaking standard care for the outcomes measured, as used in the T2Diet study [53]. Dietary quality could also be assessed to understand whether this is an independent factor of adherence and outcome measures. This may require the development of a specific dietary quality assessment tool for VLCKDs and LCDs.

## 6. Conclusions

T2D was previously considered chronic and progressive, with worsening glycaemic control directly attributable to the gradual decline in function and eventual failure of the pancreatic beta cells [82]. However, the results of this study demonstrate that the use of a 6-month mHealth low-carbohydrate dietary intervention can help people with T2D achieve better glycaemic control, reduction in cardiometabolic risk, weight loss, and improvement in liver function and can be achieved with minimal additional health care sector resources and reductions in medication use. Future analysis will assess the effect of the Defeat Diabetes mHealth app over a longer period and reassess the impact on clinical markers after 12 months of intervention and seek to provide further evidence regarding the sustainability of the approach and the likelihood of T2D remission.

In conclusion, the current study reported significant improvements in glycaemic control (i.e., HbA1c and FBG levels), with 85% of participants reducing their HbA1c levels over this time. We also reported a reduction in the cardiometabolic risk profile, as determined by serum triglyceride levels, total cholesterol to HDL-c, triglyceride to HDL-c, and waist to height ratios. In addition, there was a reduction in liver enzymes and body weight over this time. These results show that the Defeat Diabetes mHealth app can provide people with the education, resources, and support to help them implement an LCD approach to manage their T2D. Thus, the mHealth app may be considered as an additional tool for health care providers to support their patients more efficiently.

## Figures and Tables

**Table 1:** Descriptive characteristics of study participants at baseline in the total sample and by gender.

	Total Sample	Male	Female	*p* Value
	(*n* = 99)	(*n* = 44)	(*n* = 55)	
Socio-demographics				
Age (years) (Mean (SD))	58.4 (11.3)	57.6 (11.2)	59.1 (11.4)	0.518
Education Level (%)				0.636
Up to Secondary	34.3	31.8	36.4	
Higher education	65.7	68.2	63.6	
Country of Birth (%)				0.129
Australia	60.6	52.3	67.3	
Overseas	39.4	47.7	32.7	
Employment Status (%)				0.031
Unemployed	5.1	0	9.3	
Casual/Part-time/Full-time	63.3	75.0	53.7 *	
Retired	31.6	25.0	37.0 *	
No. of People in Household (%)				0.637
One person	18.2	16.3	20.0	
Two or more people	80.8	83.7	80.0	
Years with Type 2 Diabetes (Median (IQR))	2.9 (6.0)	2.0 (5.1)	4.0 (7.0)	0.017
Time since diagnosis (%)				0.003
Up to six years	68.7	79.5	60.0	
Six or more years	31.3	20.5	40.0	
Co-existing medical conditions (%)				0.387
None	22.2	18.2	25.5	
One or more	77.8	81.8	74.5	
Diabetes Medications (%)				0.485
No Medication	28.3	31.8	25.5	
Medications	71.7	68.2	74.5	
Antihypertensive Medications (%)				0.857
No Medication	46.5	45.5	47.3	
Medications	53.5	54.5	52.7	
Cholesterol-Lowering Medications (%)				0.821
No Medication	44.4	43.2	45.5	
Medications	55.6	56.8	54.5	
IPAQ Activity Level (%)				0.495
Low	26.3	20.5	30.9	
Medium	41.4	45.5	38.2	
High	32.3	34.1	30.9	

*p* values that compare continuous variables between genders are derived from the independent sample *t*-test or the non-parametric Mann–Whitney test, i.e., as per the normality of their distribution. The *p* values that compare categorical variables are derived from the chi-square test. *p* < 0.05 in the pairwise comparisons in proportions between genders. * indicates statistically significant pairwise differences between males and females.

**Table 2:** Changes in the dietary intake of energy and macronutrients from baseline to 6 months of follow-up in the total sample and by gender.

	Baseline			6-Month Follow-Up			6-Month Change			
Dietary Intake of	*n*	Mean	SD	*n*	Mean	SD	Mean Change	(95% CI) Lower	(95% CI) Upper	*p* Value
Energy (kJ/day)										
Total Sample	99	7969	2378	91	6667	1869	−1301	−1917	−685	<0.001
Males	44	8791	2357	37	7542	1857	−1249	−2201	−297	0.01
Females	55	7311	2202	54	6068	1640	−1243	−1981	−504	0.00
CHO (%kJ/day)										
Total Sample	99	32	10	91	18	10	−14	−17	−11	<0.001
Males	44	29	12	37	17	9	−12	−17	−7	<0.001
Females	55	34	8	54	19	10	−16	−19	−12	<0.001
Protein (%kJ/day)										
Total Sample	99	22	6	91	28	7	6	4	8	<0.001
Males	44	23	7	37	29	7	6	2	9	<0.001
Females	55	21	4	54	27	8	6	4	8	<0.001
Total fat (%kJ/day)										
Total Sample	99	40	7	91	49	9	9	6	11	<0.001
Males	44	41	8	37	48	8	7	4	11	<0.001
Females	55	40	7	54	49	9	10	6	13	<0.001
SAT fat (%kJ/day)										
Total Sample	99	14	4	91	18	5	4	3	5	<0.001
Males	44	13	3	37	17	4	4	2	5	<0.000
Females	55	15	5	54	19	5	4	2	6	<0.001
MONO fat (%fat/day)										
Total Sample	99	44	7	91	44	6	0	−1	2	0.71
Males	44	46	6	37	46	6	0	−3	3	0.94
Females	55	42	7	54	43	6	1	−2	3	0.60
POLY fat (%fat/day)										
Total Sample	99	17	6	91	14	5	−3	−4	−1	<0.001
Males	44	19	6	37	15	4	−4	−6	−2	<0.001
Females	55	16	5	54	14	5	−2	−4	0	0.04
Fibre (g/day)										
Total Sample	99	21	8	91	19	8	−3	−5	0	0.02
Males	44	23	9	37	19	9	−4	−8	0	0.07
Females	55	20	7	54	18	8	−2	−5	1	0.17

*p* values were derived from the independent sample *t*-test and indicate the statistical significance of the changes from baseline to 6 months of follow-up. CHO, carbohydrate; SAT, saturated; MONO, monounsaturated; POLY, polyunsaturated; SD, standard deviation from the mean; CI, confidence interval.

**Table 3:** Changes in diabetes-related blood markers, liver enzymes, waist circumference, and cardiometabolic risk ratios from baseline to 6 months of follow-up in the total sample and by gender.

	Baseline			6-Month Follow-Up			6-Month Change			
	*n*	Mean	SD	*n*	Mean	SD	Mean Change	(95% CI) Lower	(95% CI) Upper	*p* Value
Diabetes blood markers										
HbA1c %										
Total Sample	99	7.7	1.3	91	6.8	0.9	−1.0	−1.3	−0.6	<0.001
Males	44	7.9	1.3	38	6.8	0.9	−1.2	−1.7	−0.7	<0.001
Females	55	7.6	1.3	53	6.7	1.0	−0.8	−1.2	−0.4	<0.001
Fasting plasma glucose mmol/L										
Total Sample	93	8.6	2.7	81	7.4	1.8	−1.3	−2.0	−0.6	<0.001
Males	41	8.3	2.2	30	7.2	1.6	−1.0	−2.0	−0.1	0.04
Females	52	8.9	3.1	51	7.5	1.9	−1.4	−2.4	−0.4	0.01
Liver enzymes										
ALT U/L										
Total Sample	97	39.7	28.8	86	30.2	16.4	−9.3	−16.3	−2.4	0.01
Males	43	40.1	28.5	36	31.5	18.5	−8.0	−18.8	2.7	0.14
Females	54	39.4	29.4	50	29.2	14.8	−10.2	−19.4	−1.0	0.03
GGT U/L										
Total Sample	95	53.3	54.0	86	34.3	24.9	−18.8	−31.4	−6.3	0.00
Males	42	58.3	59.2	36	37.2	24.5	−21.4	−42.6	−0.3	0.05
Females	53	49.4	49.8	50	32.2	25.2	−17.4	−32.9	−1.9	0.03
Waist circumference cm										
Total Sample	96	113.6	15.3	86	108.8	14.6	−4.6	−8.9	−0.2	0.04
Males	42	114.7	15.4	33	112.6	16.0	−2.2	−9.5	5.1	0.55
Females	54	112.8	15.3	53	106.5	13.2	−6.3	−11.7	−0.9	0.02
Cardiometabolic risk ratios										
Total Cholesterol/HDL-c										
Total Sample	98	4.16	1.26	85	3.76	1.23	−0.38	−0.70	−0.05	0.03
Males	44	4.29	1.30	37	3.93	1.26	−0.28	−0.78	0.22	0.26
Females	54	4.06	1.24	48	3.62	1.21	−0.44	−0.89	0.00	0.52
TRIG/HDL-c (mmol/L/mmol/L)										
Total Sample	98	2.00	1.74	85	1.48	1.18	−0.50	−0.92	−0.08	0.02
Males	44	2.45	2.27	37	1.72	1.45	−0.70	−1.56	0.16	0.11
Females	54	1.64	1.03	48	1.29	0.90	−0.35	−0.70	0.00	0.05
Waist to Height ratio										
Total Sample	96	0.67	0.09	86	0.65	0.08	−0.02	−0.05	0.00	0.05
Males	42	0.65	0.09	33	0.65	0.09	−0.01	−0.05	0.04	0.81
Females	54	0.69	0.08	53	0.65	0.07	−0.04	−0.07	−0.01	0.01

*p* values were derived from the independent sample *t*-test and indicate the statistical significance of the changes from baseline to 6 months of follow-up. Statistical analyses were adjusted for age and gender (only in the case of total sample). SD, standard deviation from the mean; CI, confidence interval.

**Table 4:** Changes in HbA1c levels by levels of weight loss overall in the total sample and by gender.

	Δ HbA1c (%)						
	≥5% of Weight Loss			<5% of Weight Loss (Including Weight Gain)			
	*n*	Mean Change	SD	*n*	Mean Change	SD	*p* Value
Total Sample	39	−1.2	1.1	50	−0.7	1.3	0.047
Males	12	−1.3	0.9	24	−1.2	1.6	0.907
Females	27	−1.2	1.2	26	−0.3	0.7	<0.001

*p* values that compare continuous variables between genders are derived from the independent sample *t*-test or the non-parametric Mann–Whitney test, i.e., as per the normality of their distribution. The *p* values that compare categorical variables are derived from the chi-square test. Note: Small sample size is the limitation for gender comparison.

**Table 5:** Adherence to Defeat Diabetes mHealth app and impact on change in HbA1c and weight loss.

				6-Month Change			
	*n*	Mean	SD	Mean Change	(95% CI) Lower	(95% CI) Upper	*p* Value
Δ HbA1c (%)				−0.7	−1.2	−0.1	0.017
≤50 g CHO per day	30	−1.4	1.4				
>50 g CHO per day	56	−0.7	1.1				
Weight Loss (kg)				−4.2	−6.2	−2.2	<0.001
≤50 g CHO per day	31	−8.0	5.2				
>50 g CHO per day	56	−3.8	4.1				
Male							
Δ HbA1c (%)				−0.5	−1.6	0.6	0.387
≤50 g CHO per day	10	−1.6	1.7				
>50 g CHO per day	24	−1.1	1.3				
Weight Loss (kg)				−3.8	−7.4	−0.1	0.044
≤50 g CHO per day	11	−7.7	6.2				
>50 g CHO per day	24	−3.9	4.3				
Female							
Δ HbA1c (%)				−0.9	−1.5	−0.3	0.005
≤50 g CHO per day	20	−1.3	1.3				
>50 g CHO per day	32	−0.4	0.8				
Weight Loss (kg)				−4.5	−7.0	−2.1	<0.001
≤50 g CHO per day	20	−8.2	4.6				
>50 g CHO per day	32	−3.7	4.0				

*p* values that compare continuous variables between genders are derived from the independent sample *t*-test or the non-parametric Mann–Whitney test, i.e., as per the normality of their distribution. The *p* values that compare categorical variables are derived from the chi-square test. Note: Small sample size is the limitation for gender comparison. CHO, carbohydrate; SD, standard deviation from the mean; CI, confidence interval.

## Data Availability

The datasets used and analysed during this study will be available from the corresponding author G.M. on reasonable request. The data are not publicly available due to privacy considerations to fulfill ethical obligations.

## Supplementary Materials

The following supporting information can be downloaded at: https://www.mdpi.com/article/10.3390/nu17060937/s1, Table S1: Changes in serum lipids, kidney function, blood pressure and anthropometric markers from baseline to 6 months of follow-up in the total sample and by gender; Table S2: Changes in weight status and glycaemic control after 6 months.
