# Supplementary material for: A 6-Month mHealth Low-Carbohydrate Dietary Intervention Ameliorates Glycaemic and Cardiometabolic Risk Profile in People with Type 2 Diabetes
Source: Nutrients. 2025 Mar 7;17(6):937. doi: 10.3390/nu17060937 (PMC11946380; doi:10.3390/nu17060937)
Supplement: Supplementary file 1 [file nutrients-17-00937-s001.zip › nutrients-3487488-supplementary.pdf]

A 6-month mHealth low-carbohydrate dietary intervention ameliorates glycaemic and cardiometabolic risk profile in people with type 2 diabetes

Supplementary Information

Author Information:

Despina Kolivas (ORCID 0000-0003-1195-0688)[1,*], Liz Fraser(ORCID 0009-0000-9108-2440)[2], Ronald Schweitzer(ORCID 0009-0005-2893-7967)[3,4], Peter Brukner(ORCID 0000-0001-9923-4461)[1], and George Moschonis (ORCID 0000-0003-3009-6675)[1,5*]

[1] School of Allied Health, Human Services & Sport, La Trobe University, Bundoora 3086, Australia

[2] Watson General Practice, 34 Windeyer Street, Watson 2602, Australia.

[3] East Bentleigh Medical Group, 873 Centre Road, Bentleigh East 3165, Australia.

[4] Department of General Practice, School of Public Health and Preventive Medicine, Monash University, Level 5, 553 St Kilda Rd, Melbourne 3004, Australia

[5] La Trobe Institute for Sustainable Agriculture & Food (LISAF), La Trobe University, Bundoora, VIC 3086, Australia

[*] Correspondence: d.kolivas@latrobe.edu.au (D.K.), g.moschonis@latrobe.edu.au (G.M.)

Table S1 *Changes in serum lipids, kidney function, blood pressure and anthropometric markers from baseline to 6 months of follow-up in the total sample and by gender.*

	Baseline			6 month follow-up			6-months Change			
	n	Mean	SD	n	Mean	SD	Mean change	(95% CI) Lower	(95% CI) Upper	P-value
**Serum lipids**										
**Total Cholesterol mmol/L**										
Total Sample	99	4.9	1.2	87	4.9	1.2	-0.1	-0.4	0.3	0.72
Males	44	4.8	1.3	37	4.8	1.4	0.1	-0.4	0.7	0.71
Females	55	5.1	1.1	50	4.9	1.1	-0.2	-0.6	0.3	0.45
**LDL-c mmol/L**										
Total Sample	93	2.8	1.1	82	2.7	1.1	-0.1	-0.4	0.2	0.58
Males	39	2.7	1.1	34	2.8	1.2	0.1	-0.4	0.6	0.61
Females	54	2.9	1.1	48	2.7	1.0	-0.2	-0.6	0.2	0.28
**HDL-c mmol/L**										
Total Sample	98	1.3	0.4	85	1.4	0.5	0.1	0.0	0.3	0.06
Males	44	1.2	0.5	37	1.3	0.3	0.1	-0.1	0.3	0.36
Females	54	1.3	0.3	48	1.5	0.7	0.2	0.0	0.4	0.10
**Triglycerides mmol/L**										
Total Sample	99	2.2	1.6	87	1.7	1.1	-0.4	-0.8	-0.1	0.02
Males	44	2.5	2.1	37	1.9	1.4	-0.6	-1.4	0.2	0.17
Females	55	2.0	0.9	50	1.6	0.8	-0.4	-0.7	0.0	0.02
**Kidney function**										
**eGFR mL/min/1.73m^2**										
Total Sample	97	81.4	12.2	88	81.9	12.5	0.6	-2.5	3.8	0.70
Males	42	81.1	13.7	37	81.7	14.3	1.1	-4.4	6.5	0.69
Females	55	81.7	11.1	51	82.0	11.1	0.3	-3.5	4.1	0.86
**Haemodynamics**										
**Systolic blood pressure mmHg**										
Total Sample	99	135	15	86	133	14	-2	-7	2	0.26
Males	44	138	15	35	133	10	-6	-12	0	0.06
Females	55	133	15	51	133	16	0	-6	6	0.99
**Diastolic blood pressure mmHg**										
Total Sample	99	83	11	86	80.0	9.9	-2.4	-5.3	0.6	0.12
Males	44	83	12	35	80.2	9.5	-2.7	-7.4	2.0	0.26
Females	55	82	10	51	79.9	10.2	-2.1	-5.9	1.8	0.29
**Anthropometry**										
**Body weight kg**										
Total Sample	99	98.0	22.2	91	92.6	21.0	-4.9	-10.7	1.0	0.10
Males	44	104.5	20.3	38	101.0	19.4	-3.3	-12.1	5.4	0.45
Females	55	92.7	22.5	53	86.6	20.2	-6.1	-14.0	1.8	0.13
**BMI kg/m^2**										
Total Sample	99	33.9	6.3	91	32.2	5.9	-1.7	-3.4	0.0	0.05
Males	44	33.3	5.8	38	32.2	5.6	-1.1	-3.6	1.4	0.39
Females	55	34.4	6.7	53	32.1	6.1	-2.2	-4.6	0.2	0.07

Table S2 *Changes in weight status and glycaemic control after 6 months*

**Weight status of participants**	**n**	**%**	**Glycaemic control**	**n**	%
Did not lose weight (or gained)	12	14	No change or increase in HbA1c	14	15
Lost weight	74	86	HbA1c reduction from baseline	77	85
**Changes in weight status**			**Changes in glycaemic control**		
Greater than or equal to 5% weight loss	41	45	HbA1c<6.5	46	51
Less than 5% weight loss (including weight gain)	50	55	HbA1c≥6.5	45	50
